# Translation of monosynaptic circuits underlying amygdala fMRI neurofeedback training

**DOI:** 10.1038/s41386-024-01944-w

**Published:** 2024-08-05

**Authors:** Lucas Trambaiolli, Chiara Maffei, Evan Dann, Claudinei Biazoli Jr, Gleb Bezgin, Anastasia Yendiki, Suzanne Haber

**Affiliations:** 1 McLean Hospital, Harvard Medical School, Boston, MA USA; 2 Department of Pharmacology and Physiology, University of Rochester, Rochester, NY USA; 3 Athinoula A. Martinos Center for Biomedical Imaging, Massachusetts General Hospital and Harvard Medical School, Boston, MA USA; 4 Center for Neurotechnology and Neurorecovery, Department of Neurology, Massachusetts General Hospital, Boston, MA USA; 5 Center for Mathematics Computation and Cognition, Federal University of ABC, Santo André, Brazil; 6 School of Biological and Behavioural Sciences, Queen Mary University of London, London, UK; 7 Neuroinformatics for Personalized Medicine lab, Montreal Neurological Institute, McGill University, Montréal, QC Canada

**Keywords:** Amygdala, Limbic system

## Abstract

fMRI neurofeedback using autobiographical memory recall to upregulate the amygdala is associated with resting-state functional connectivity (rsFC) changes between the amygdala and the salience and default mode networks (SN and DMN, respectively). We hypothesize the existence of anatomical circuits underlying these rsFC changes. Using a cross-species brain parcellation, we identified in non-human primates locations homologous to the regions of interest (ROIs) from studies showing pre-to-post-neurofeedback changes in rsFC with the left amygdala. We injected bidirectional tracers in the basolateral, lateral, and central amygdala nuclei of adult macaques and used bright- and dark-field microscopy to identify cells and axon terminals in each ROI (SN: anterior cingulate, ventrolateral, and insular cortices; DMN: temporal pole, middle frontal gyrus, angular gyrus, precuneus, posterior cingulate cortex, parahippocampal gyrus, hippocampus, and thalamus). We also performed additional injections in specific ROIs to validate the results following amygdala injections and delineate potential disynaptic pathways. Finally, we used high-resolution diffusion MRI data from four *post-mortem* macaque brains and one in vivo human brain to translate our findings to the neuroimaging domain. Different amygdala nuclei had significant monosynaptic connections with all the SN and DMN ipsilateral ROIs. Amygdala connections with the DMN contralateral ROIs are disynaptic through the hippocampus and parahippocampal gyrus. Diffusion MRI in both species benefitted from using the ground-truth tracer data to validate its findings, as we identified false-negative ipsilateral and false-positive contralateral connectivity results. This study provides the foundation for future causal investigations of amygdala neurofeedback modulation of the SN and DMN through these anatomic connections.

## Introduction

Functional Magnetic Resonance Imaging (fMRI) neurofeedback modulates specific brain regions in real time through self-elicited mental strategies [1]. It is considered a potential therapeutic approach for several psychiatric disorders, including depression and anxiety [2–4]. Comparing resting-state functional connectivity (rsFC) before and after neurofeedback provides insights into which brain networks support the long-lasting effects of neuromodulation [5, 6]. However, fMRI is an indirect method for connectivity analysis. Delineating the hard-wired, monosynaptic connections that underlie these rsFC results will lead to: (i) identifying the circuitries underlying neurofeedback mechanisms; (ii) probing those circuits with human and animal models; (iii) guiding personalized neurofeedback protocols. This study aims to determine the anatomic, monosynaptic connections underlying rsFC changes elicited by neurofeedback modulation. Given the exploratory approach of this study, we did not include neurofeedback training in new animals. We used existing anatomic tracing data and high-resolution diffusion MRI (dMRI) data in macaques and humans to determine the most likely monosynaptic circuitry related to rsFC changes following neurofeedback intervention.

We focused on rsFC changes following neurofeedback using autobiographical memory recall to up-regulate the amygdala [7, 8]. The current hypothesis is that clinical improvement following amygdala neurofeedback results from its modulation of two large-scale networks: the salience and the default mode networks (SN and DMN, respectively) [8–12]. Although connections between the amygdala and SN and DMN nodes have been described [13–34], these are large regions with several subdivisions, each with different connectivity patterns [15, 23, 24, 27, 29, 30]. Here, we combined multi-modal multi-species data to delineate the anatomical circuits connecting the amygdala and the specific DMN and SN sublocations (or regions-of-interest - ROIs) modulated by amygdala neurofeedback [5, 12, 35]. Specifically, we: *1*. Identified the equivalent ROIs in the non-human primate (NHP) brain. *2*. Analyzed the anatomical connections between each sublocation and the amygdala. *3*. Tested whether the same connections could be identified using submillimeter (500 μm) ex vivo dMRI tractography data in NHP. *4*. Tested whether these connections could also be identified in human submillimeter (760 μm) in vivo dMRI.

## Methods

### Step 1: Translating rsFC ROIs from the human to the NHP brain

We extracted the rsFC ROIs from all three studies [5, 12, 35] that used seed-based connectivity analysis to evaluate an fMRI neurofeedback protocol based on positive autobiographical memory recall to up-regulate the left amygdala. There were 16 ROIs (Supplementary Table 1) in which rsFC with the left amygdala changed after neurofeedback, including some, but not all, ROIs of the SN (dorsal anterior cingulate cortex - dACC, anterior insula – AI, and lateral prefrontal cortex - LPFC) and DMN (middle frontal gyrus - MFG, temporal pole - TP, hippocampus, parahippocampal gyrus – PHG, precuneus, posterior cingulate cortex – PCC, angular gyrus and thalamus). The coordinates of these ROIs were transformed to the MNI space based on Lancaster et al. [36].

We used the “Regional Map” parcellation, a standard for cross-species comparisons [37], to identify equivalent ROIs across NHPs and humans. Details about this parcellation are included in the *Supplement*. The equivalent ROIs in the macaque brain were manually placed according to homologous parcels [37], cytoarchitectonic areas [38, 39], and morphological landmarks [40] (please refer to Supplementary Table 1 for a detailed description of each ROI location). Importantly, in this study, we used human terminology when referring to the NHP ROIs (e.g., although we list ROIs in the “angular gyrus” and “middle frontal gyrus” macaques don’t have these gyri in the strict sense).

### Step 2: Identification of anatomical connections using NHP tract-tracer data

We selected 12 cases – different injections in different animals (four *Macaca mulatta*, four *Macaca fascicularis*, and four *Macaca nemestrina*) - from the HaberLab collection of brains with bidirectional tracer injections placed throughout cortical and subcortical areas of adult male monkeys. The University Committee on Animal Resources from the University of Rochester approved all tracer experiments, and animal care followed the National Guide for the Care and Use of Laboratory Animals.

For the seven injections in the amygdala (Supplementary Fig. 1A), we evaluated connections with each SN and DMN ROIs from Supplementary Table 1. We used the additional five injections in specific ROIs (anterior insula, lateral precuneus, hippocampus, posterior cingulate cortex, and thalamus) to validate the connectivity patterns with the amygdala. The surgical and histological procedures are detailed in the *Supplement*.

We charted the retrogradely labeled cells in the ROIs under light-field microscopy at 20 x (Supplementary Fig. 1C) [41–43]. We quantified the strength of inputs to the amygdala based on the density of cells per mm^2^ [41]. We used dark-field microscopy under 1.6 x, 4 x, and 10 x objectives to outline dense or light axon terminal projections. We labeled condensed groups of fibers visible at 1.6 x with discernible boundaries as ‘dense projections’ and groups of fibers where individual terminals could be discerned as ‘light projections’ (Supplementary Fig. 1C) [41, 43]. We quantified the strength of outputs from the amygdala based on the weighted density of axon terminal projections per mm^2^, with dense and light projections receiving weights of 1 and 0.5, respectively.

### Step 3: Identification of anatomical connections using NHP tractography data

The NHP postmortem submillimeter dMRI (500 μm) data was collected from four adult animals, with a total scan time of 47 h per brain (MRI acquisition and preprocessing details can be found in the *Supplement*). The left amygdala was extracted from the D99 macaque atlas [44]. The location of each rsFC ROI was identified as a single point on the F99 macaque brain. These point coordinates were mapped to each individual brain using the transforms from the registration described in the *Supplement*. For each point, we found its nearest voxel along the white-gray matter boundary. Spherical ROIs were defined with a 1.5 mm radius around these points. Streamlines connecting the left amygdala and each ROI included in this analysis were manually dissected using Trackvis (v.0.6.1; http://www.trackvis.org). Streamlines connecting the left amygdala with each ROI were filtered to only include those streamlines ending or originating inside the amygdala mask.

### Step 4: Human tractography analysis

We used submillimeter-resolution dMRI (760 μm) data from a publicly available and pre-processed dataset [45]. Processing followed similar steps to those previously described for the NHP data (see details in the *Supplement*). Streamlines connecting the left amygdala and each ROI from Table 1 were manually dissected using Trackvis. We created a sphere around the center coordinates reported in the reference studies matching the amygdala ROI used as the neurofeedback target [11] and connectivity seed [5, 12]. Since the reported 7 mm radius included only gray matter, we used a 10 mm radius to include the surrounding white matter. For all other cortical ROIs, we used spheres with a 7 mm radius centering at the border between white and gray matter closest to the ROI coordinates.

## Results

### Step 1: Cross-species ROIs selected for this study

**Fig. 1:**
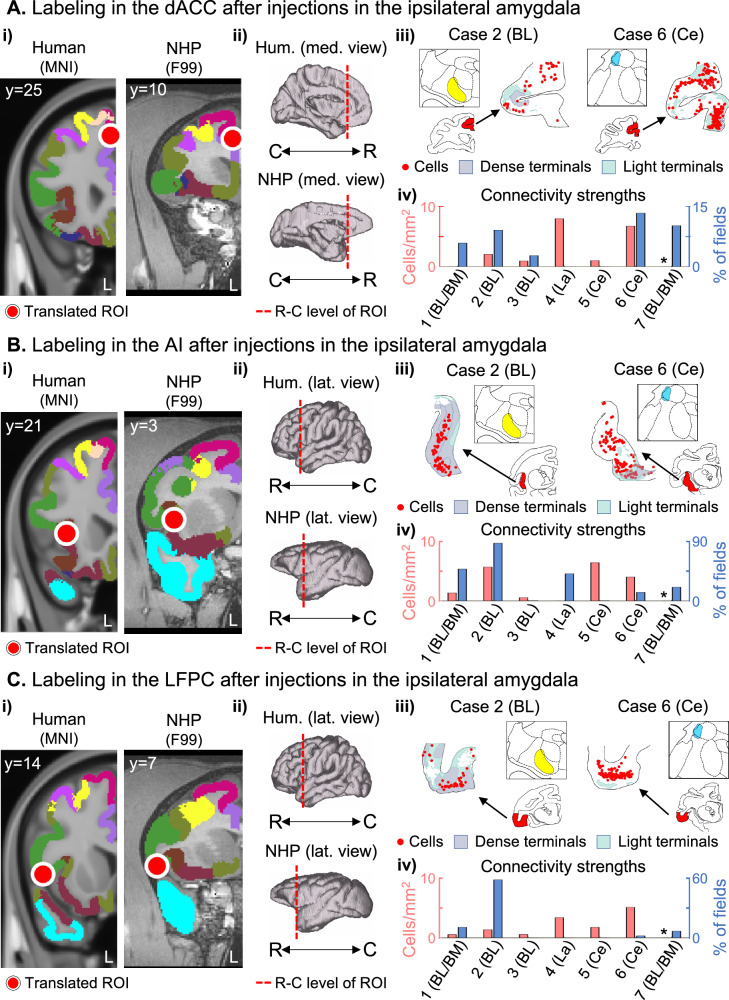
Amygdala connections with the ipsilateral SN nodes. **A.i** Red circles indicate the peak location of the rsFC changes after amygdala neurofeedback for the dACC in the human MNI template and the homologous location in the macaque F99 template. **A.ii** 3D models represent the rostro-caudal location of coronal slices from each node. **A.iii** The square box shows the injection site for two representative cases (in different animals), and the schematic coronal sections highlight the location with connectivity chartings in red. Individual cells are shown as red dots, dense/moderate terminals as light blue shaded areas, and diffuse terminals as light green shaded areas. **A.iv** Connectivity strengths for each case are shown in bar plots, with the first *y*-axis (red) representing the strength of the ROI inputs to the amygdala and the second *y*-axis (blue) representing the strengths of amygdala outputs to the ROI. *Case 7 had only anterograde transport and no input strength value. Additional cases are shown in Supplementary Fig. 2. The same organization followed for ROIs in the AI **B** and LPFC **C**. BL basolateral nucleus, BM basomedial nucleus, C caudal, Ce central nucleus, La lateral nucleus, R rostral.

We identified the 16 ROIs in the NHP brain based on anatomical, morphological, and cytoarchitectonic criteria. The resulting center coordinates in the F99 space and criteria are listed in Supplementary Table 1. The SN ROIs in the NHP brain included the ipsilateral (left hemisphere) dACC (at the genu of the corpus callosum including area 24 and 6/32, Fig. 1Ai–ii), AI (at the rostral portion of the circular sulcus including area AI, Fig. 1Bi–ii), and LPFC (caudal area 47/12O, 44 and ProM at the dorsal lip of the rostral part of lateral fissure Fig. 1Ci–ii).

**Fig. 2:**
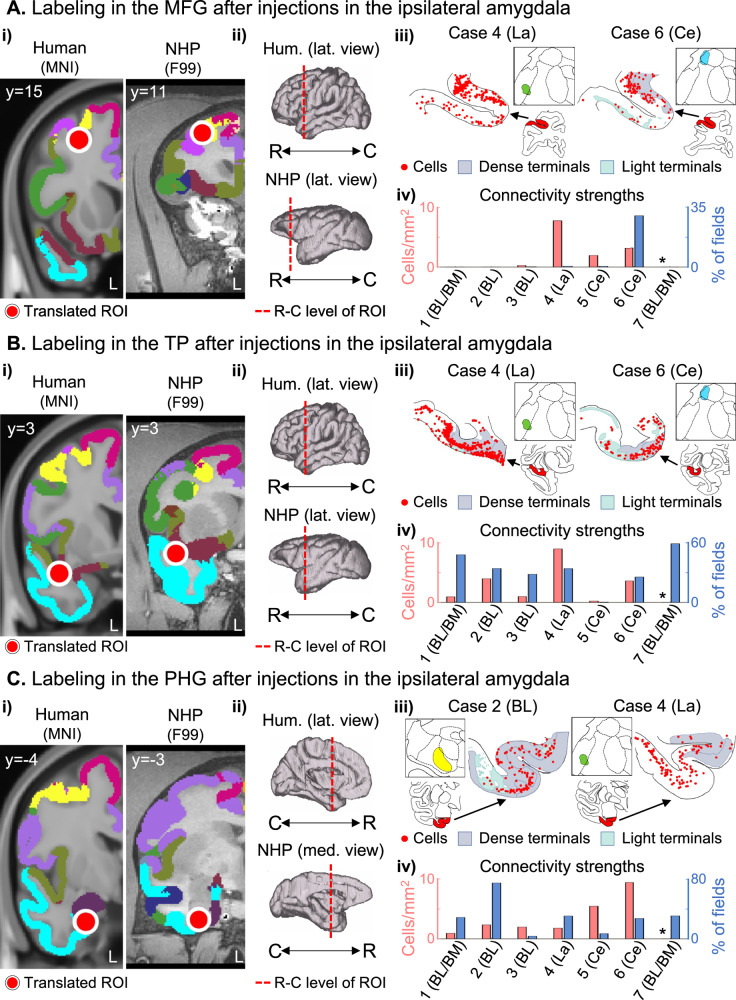

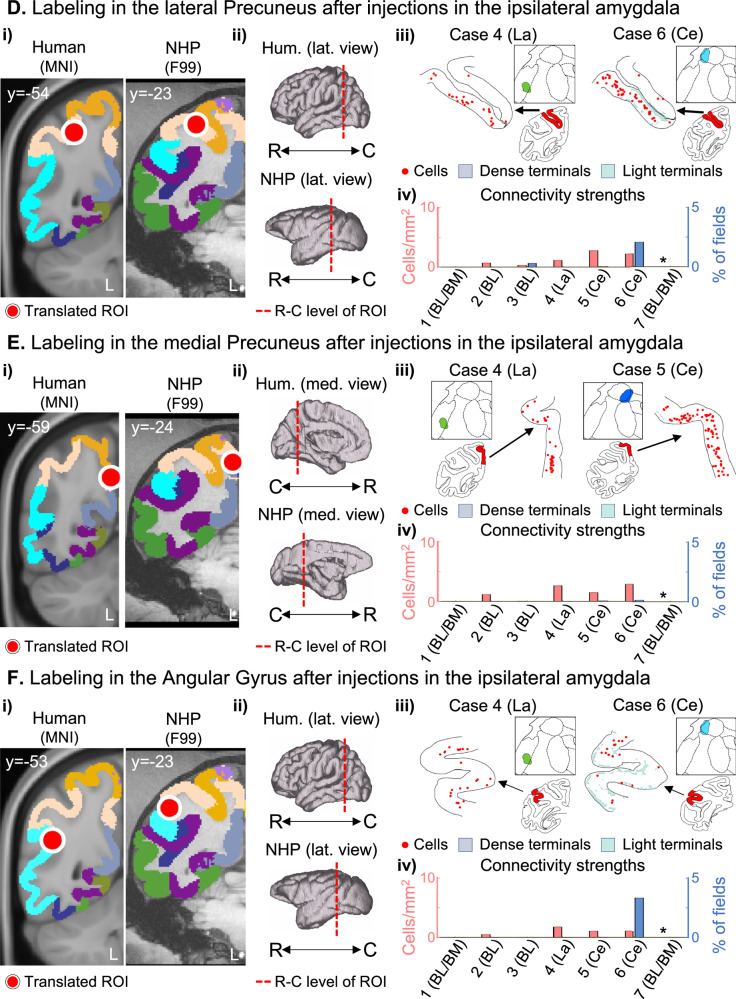
Amygdala connections with the ipsilateral DMN nodes. The same organization of Fig. 1 is used to show amygdala connections (representative cases from different animals) with the Middle Frontal Gyrus **A**, Temporal Pole **B**, Parahippocampal Gyrus **C**, Lateral Precuneus **D**, Medial Precuneus **E** and Angular Gyrus **F**. Additional cases are shown in Supplementary Fig. 3. BL basolateral nucleus, BM basomedial nucleus, C caudal, Ce central nucleus, La lateral nucleus, R rostral.

The ipsilateral ROIs of the DMN included the MFG (at the ventral bank of the superior arcuate sulcus, in the border of areas 8AB, 8B, and 9/46D - Fig. 2Ai-ii) in the frontal cortex. In the temporal cortex, the TP at the ventral bank of the circular sulcus including areas IPro and TPPro (Fig. 2Bi-ii) and PHG (dorsal and medial to the rhinal fissure, at the border of areas EOI, ELR, and ER - Fig. 2Ci-ii). Finally, in the parietal cortex, ROIs included the Lateral Precuneus (at the lip of the ventral bank of the intraparietal sulcus, including areas POaE/LIPE and PG - Fig. 2Di-ii), Medial Precuneus (at the ventral bank of the posterior cingulate sulcus, including areas PGm and 31 - Fig. 2Ei-ii), and Angular Gyrus (AG, in the caudal portion of the lateral fissure, including the border of areas PGOp, ReI, and Tpt - Fig. 2Fi-ii).

The DMN ROIs in the contralateral hemisphere included the MFG (at the ventral bank of the principal sulcus, including areas 9/46V and 46V) in the frontal cortex. In the temporal cortex, TP (at the dorsolateral portion of the anterior temporal lobe, in area TPPro extending to ST1 – Supplementary Fig. 5A-right) and PHG (at the lateral bank of the rhinal fissure, including areas TLR/R36 and TH - Supplementary Fig. 5A-center). In the parietal cortex, PCC (area 23 and 30 in the cingulate gyrus – Supplementary Fig. 5B) and Medial Precuneus (dorsal bank of the cingulate sulcus, in area 31 extending to area PECg). Subcortical ROIs include the dorsal Hippocampus (Supplementary Fig. 5A-left) and Thalamus (Supplementary Fig. 5C).

### Step 2: Anatomical connections identified using NHP tract-tracer data

**Fig. 3:**
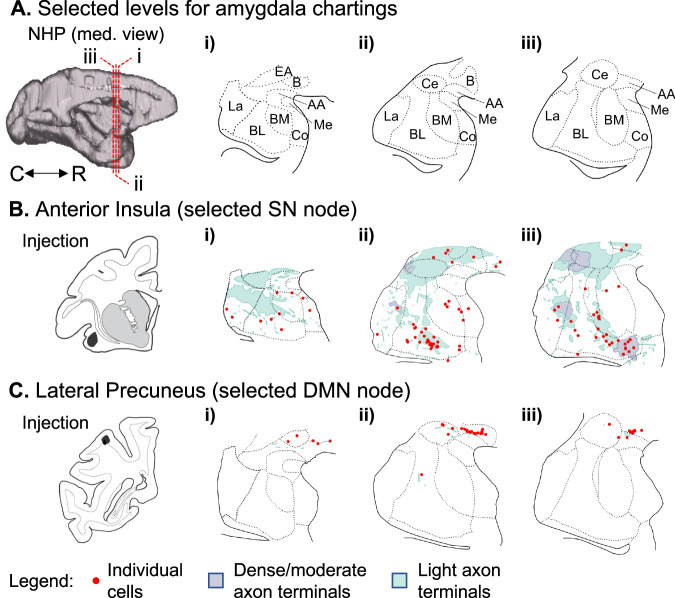
Validation of amygdala connections after cortical injections. **A** 3D representation of the three rostro-caudal levels (i-iii) used to chart cells and terminals in the amygdala, and the respective coronal slices with cytoarchitectonic divisions based on the Paxinos atlas [38]. Labeling of cells (red dots), and dense/moderate (light blue) and diffuse (light green) terminal fields in the amygdala after bidirectional tracer injections (in different animals) in regions homologous to the Anterior Insula **B** and Lateral Precuneus **C** regions with resting-state functional connectivity changes after amygdala neurofeedback. AA anterior amygdaloid area, BL basolateral nucleus, BM basomedial nucleus, C caudal, Ce central nucleus, La lateral nucleus, R rostral.

Bidirectional tracer injections in the amygdala showed monosynaptic connections with the ipsilateral dACC, AI, and LPFC ROIs within the SN Fig. 1 (additional cases in Supplementary Fig. 2). Importantly, the basolateral (BL), lateral (La), and lateral central (Ce, case 6) amygdala nuclei, had bidirectional connections with these three ROIs. To validate the existence and specificity of the observed connections, we identified a small bidirectional tracer injection in the AI (Fig. 3B) that showed bidirectional connectivity patterns spread along all amygdala nuclei, consistent with the results in Fig. 1B.

The amygdala was also interconnected with the ipsilateral DMN sublocations modulated by neurofeedback (Fig. 2 and Supplementary Fig. 3). ROIs closer to the amygdala (TP and PHG, Fig. 2B-C) were bidirectionally connected with all injection locations. The MFG (Fig. 2A), precuneus (Fig. 2D-E), and angular gyrus (Fig. 2F) connectivity strengths were generally weaker (reduced cell density) than those from the other ROIs. These four DMN ROIs showed dense labeling with La and Ce injection sites, scarce labeling with BL injections (Cases 2 and 3), and no connections with the injection in BL/BM (Case 1). Cell labeling in the lateral precuneus ROI was mostly on the intraparietal sulcus, including the deep lateral and medial banks, beyond the ROI extension. A validation injection in the lateral precuneus (Fig. 3C) showed concentrated labeling in the dorsal bank of the amygdala, including the Basal and Ce nuclei. Although this case is lateral to the original ROI, these results are partially consistent with those observed in the amygdala injections (Fig. 2D), except for the lack of labeling in the La nucleus. Importantly, labeling in parietal structures (precuneus and angular gyrus) after injections in the amygdala and labeling in the amygdala after injection in the precuneus showed predominantly retrograde labeling.

Our data showed sparse monosynaptic connections from the left amygdala to the contralateral hemisphere and no connections with the specific contralateral DMN ROIs. Importantly, amygdala neurofeedback is also associated with changes in the hippocampus and parahippocampal gyrus (PHG) [11, 12, 46–48], regions anatomically interconnected with the amygdala [14, 26, 28]. Thus, we evaluated if the amygdala connected with the contralateral DMN ROIs through the hippocampus and PHG. Supplementary Fig. 4B shows anatomical labeling in the left hippocampus and PHG for injections in the left amygdala. Briefly, all cases presented labeling in the amygdalohippocampal area. BL and La injections showed labeling in rostral CA1’ and CA3 subfields and dense labeling in the transition between the subiculum and prosubiculum (ProS) fields, extending to areas 35, 36, and TF in the PHG. A similar but weaker pattern is also observed in Ce (Case 5). A validation injection including CA1, ProS, and Subiculum in the hippocampus to areas 35, 36, and TF in the PHG (Supplementary Fig. 4C) showed spatial labeling in the amygdala consistent with those observed in Supplementary Fig. 4B.

Supplementary Fig. 5 illustrates connections between the left hippocampus and left PHG, and the contralateral DMN ROIs (right hemisphere). The injection in the hippocampus and PHG showed anatomical connections with the contralateral hippocampus, PHG, and TP nodes of the DMN (Supplementary Fig. 5A). However, this injection did not include all structures in the hippocampus and PHG. To evaluate if the remaining contralateral DMN nodes listed in Supplementary Table 1 connected with other hippocampal and PHG subnuclei, we placed two additional injections in two of these nodes: the right PCC (Supplementary Fig. 5B) and right thalamus (Supplementary Fig. 5C). We observed axon terminal and cell labeling in the left hippocampus and PHG, respectively.

### Step 3: Anatomical connections identified with NHP tractography

**Fig. 4:**
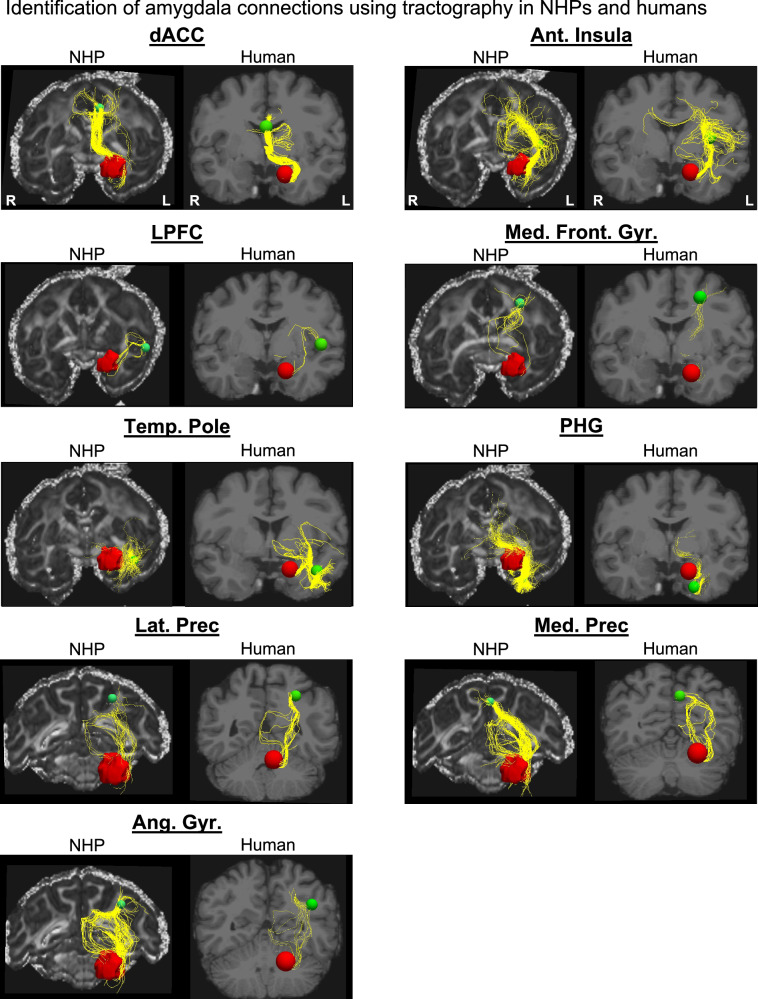
Identification of amygdala connections using dMRI tractography in NHPs and humans. Reconstruction of streamlines (yellow) connecting the amygdala (red) with all ipsilateral nodes (green) within the SN and DMN. For each node, results from one NHP brain are shown on the left, and results from the human brain on the right.

Using submillimeter dMRI tractography, we correctly identified connections between the left amygdala and all ipsilateral ROIs (Fig. 4 shows results from one animal and Supplementary Figs. 6–8 from additional animals). Importantly, inconsistent with the tracer data, connections between the amygdala and the MFG and LPFC were among those with the fewest streamlines.

Tractography data also showed streamlines connecting the left amygdala with several contralateral ROIs, disagreeing with the tracer data. We compared the tractography and the tracer data at different locations along these tracts to identify where tractography errors occurred. Supplementary Fig. 7A shows two sets of streamlines erroneously connecting the left amygdala and the right medial precuneus in one representative case. After leaving the amygdala, the anterior streamlines follow the same direction as the amygdalofugal fibers (Supplementary Fig. 9B-C). However, posteriorly, these streamlines enter the fornix (Supplementary Fig. 9D), which is inconsistent with the results from the tracer data. The posterior false positive connection follows the same trajectory as the stria terminalis observed in the tracer data (Supplementary Fig. 9E–G). Similar to the anterior false positive, streamlines erroneously follow through the fornix to the contralateral hemisphere.

### Step 4: Anatomical connections identified with human tractography

Using submillimeter human dMRI tractography, we successfully identified connections between the left amygdala and all ipsilateral ROIs. Amygdala connections with regions such as the dACC, AI, PHG, TP, and Precuneus showed the same clear tracts with dense concentrations of streamlines (Fig. 4) as observed in the NHP dMRI tractography data. Consistent with the NHP dMRI tractography data, amygdala-LPFC, and amygdala-MFG connections presented fewer, sparser streamlines than other connections. Similar to tractography results in NHP, false positive connections were also identified connecting the left amygdala with contralateral ROIs (e.g., streamlines traveling contralaterally through the fornix, Supplementary Fig. 9A).

## Discussion

### Summary

**Fig. 5:**
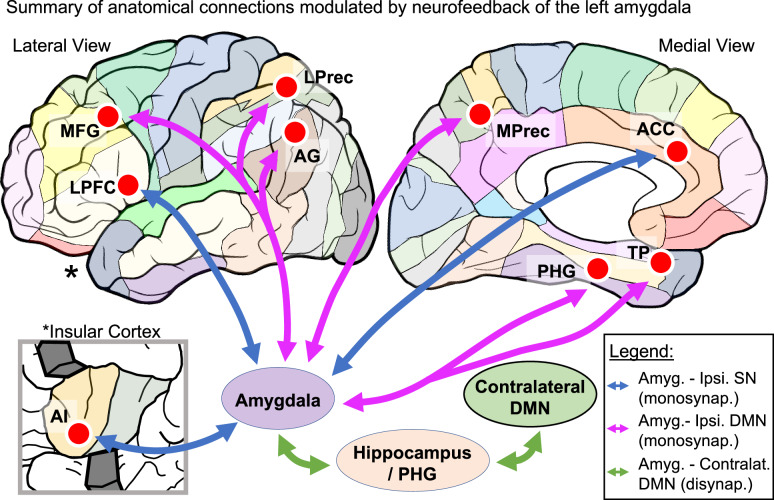
Summary of anatomical connections modulated by neurofeedback of the left amygdala. Representation of the specificity of ROIs in the left hemisphere (red circles) overlapping major functional regions (colored parcellation). Blue and pink arrows represent monosynaptic connections from the amygdala to the SN and DMN ipsilateral ROIs, respectively. Green arrows show the disynaptic connections with the DMN contralateral ROIs through the hippocampus and PHG.

The current mechanistic hypothesis of amygdala neurofeedback is that the amygdala re-directs attention toward salient positive stimuli during self-referential processing, reducing rumination and improving forward-thinking [8, 49]. These processes may occur via increased activation and functional connectivity changes in SN and DMN nodes [8–11, 46]. Neuroanatomical homologies [50, 51], including homologous SN [52] and DMN [53, 54] networks in the macaque brain, allow for a deeper delineation of these circuits involved in neurofeedback using NPHs. Previously, the NHP literature showed that the amygdala is anatomically interconnected with the large regions of the SN and DMN nodes [15, 16, 19, 20, 55]. We provide tracer and dMRI evidence that the amygdala has monosynaptic anatomical connections with specific locations within the SN and DMN ROIs relevant for neurofeedback results (Fig. 5). We also show that amygdala hard-wiring with contralateral DMN ROIs is likely disynaptic through its connections with the adjacent hippocampus and PHG [14, 26, 28], two regions highly active during amygdala neurofeedback training [11, 46, 47]. This circuit delineation allows for new mechanistic interpretations of how the amygdala interactions with the SN and DMN are associated with lasting clinical effects after neurofeedback [8]. Moreover, it may lead to future neurofeedback studies probing those mechanisms.

### Amygdaloid connections to the ROIs within the SN and DMN

Amygdala connections to SN nodes within the frontal and insular cortices are patchy and terminate in precise areas within each region [15, 19, 20, 22–24, 27, 29, 30]. Our results show that SN ROIs identified in previous neurofeedback studies fall within these patches. These monosynaptic connections support the proposed role of amygdala neurofeedback in re-directing attention toward specific salient stimuli [8, 49]. Previous NHP studies support that the amygdala works closely with the SN during salience processing [56–58]. E.g., local stimulation of the amygdala modulates the activity of the ACC and insular ROIs of the SN [59], reinforcing the potential of amygdala interactions with this network through its connections. Brain imaging and lesion studies in humans also highlighted the relevance of the amygdala and its connections in processing emotionally salient stimuli [60–64].

Amygdala-DMN connections are less precise. E.g., the amygdala has strong and widely distributed connections with the TP, Thalamus, Hippocampus, and PHG [13, 14, 17, 21, 22, 25, 26, 28–30]. However, connections with the MFG, PCC, and Precuneus are weaker and more restricted [15, 16, 18, 19, 29, 31]. Our data also showed that ipsilateral ROIs of the DMN are mainly connected with the central and basal nuclei. These nuclei are central for processing fear and anxiety [65–67], which are mediated by amygdala connections with regions processing context-specific aspects of the stress response [68, 69]. Modulation of fear and stress may play an essential role in worry and rumination, symptoms correlated with amygdala rsFC with some DMN ROIs, including the MFG and precuneus [70].

Our data showed no monosynaptic connections from the amygdala to the contralateral ROIs, consistent with previous studies [71]. However, the amygdala is tightly linked with the ipsilateral hippocampus and PHG [14, 26, 28], which are connected to the contralateral structures [72–74]. Importantly, all studies included in our analysis [5, 12, 35] used a protocol based on positive autobiographical memory recall to up-regulate the BOLD signal of the left amygdala [11]. Neurofeedback studies using this protocol reported the hippocampus and PHG coactivation during the neurofeedback training task [11, 46, 47], increased functional connectivity between the left amygdala and left hippocampal/PHG structures [11, 12], and increased gray matter volume of hippocampal subfields [48]. Complementarily, neurofeedback targeting the up-regulation of the left hippocampus during autobiographical memory recall also leads to co-activation of the amygdala and increased amygdala-hippocampus functional connectivity [75]. Together with our anatomical delineation, these results suggest that rsFC changes with the contralateral DMN ROIs could be explained via amygdala-hippocampal projections.

### Other relevant nodes for the amygdala neurofeedback circuitry

The ROIs listed in this study are specific to some but not all nodes of the SN and DMN. E.g., regions like the ventral striatum (SN node) and vmPFC (DMN node) did not change rsFC with the amygdala after neurofeedback. These regions are known to be anatomically interconnected with the amygdala [15, 23, 24, 27, 29, 30, 32–34]. Although not identified in the rsFC studies, additional evidence suggests these regions are relevant during the neurofeedback task. The ventral striatum is highly active during neurofeedback reward processing [1]. Additionally, amygdala connectivity with the vmPFC changes during neurofeedback training [11, 76]. Thus, amygdala neurofeedback may be associated with a broader modulation of the SN and DMN through anatomical connections.

Other protocols targeting the amygdala provide additional insights into other relevant nodes of the neurofeedback circuitry. A recent meta-analysis [8] evaluated potential networks engaged during successful down-regulation of amygdala activity. However, in their sample, participants did not receive any cognitive instructions. They had to develop strategies while presented with visual stimuli of different complexity levels (e.g., triggering figures [77] or VR environment [78]). Successful regulators showed enhanced activities within the executive control network (ECN) regions, including the lateral occipitoparietal and supplementary motor areas, alongside decreased activities in several regions. Although the protocol and the time points evaluated in their analysis differ from those focused here, our pipeline could be extended to delineate common anatomical circuitries underlying multiple amygdala neurofeedback approaches.

### Neuroanatomical basis of clinical effects

The studies providing the ROI coordinates [5, 12, 35] are follow-up investigations from original trials with patients with depression [46, 79] or PTSD [80]. These patients showed significant clinical improvement and reduced symptoms after neurofeedback [46, 79, 80]. Notably, around 30% of patients with depression reached remission levels at the primary endpoint [79]. These clinical effects correlated with the normalization of rsFC over the days following the neurofeedback training [5]. These clinical effects of fMRI neurofeedback training are likely associated with rebalancing abnormal functional connections.

Neurofeedback is a complex task, and rsFC changes likely result from the combined direct modulation of the amygdala and comprehensive strategy adjustments [81]. Our results and previous studies [82] show the bi-directionality of amygdala connections with the SN and DMN, suggesting both bottom-up and top-down pathways involved in the clinical improvements. From a bottom-up perspective, monosynaptic connections may allow the amygdala to modulate the ROIs of the SN and DMN quickly during neurofeedback sessions. A similar process is observed during focal stimulation of the amygdala [59, 83, 84]: after systematic reinforcement, changes in these functional connections are sustained beyond the task and observed at rest. During training, participants must also identify and optimize the best mental strategies to modulate the amygdala. This process involves high-order thinking and engages top-down control, similar to what is observed in psychotherapy [85]. In fact, a recent study showed that amygdala neurofeedback enhances the effects of cognitive-behavioral therapy in patients with depression [86], reinforcing the idea that both approaches involve similar pathways. Thus, the anatomical delineation provided in this study is essential to further test these top-down and bottom-up mechanisms of amygdala neurofeedback and further complement or potentialize the action of traditional therapeutic interventions in psychiatry.

### Technical considerations

Anatomical tract-tracing is the gold standard method for delineating connections in the primate brain [87]. However, our tracer data showed inconsistent labeling between the amygdala and lateral precuneus. In both cases, only retrograde labeling was identified. Proper tracer labeling in long-distance pathways may require up to 5 weeks of transport time [88, 89], while our cases were perfused after two weeks. Thus, a possible explanation is that the anterograde transport may need longer transport time to show labeling in long-distance connections. These transport characteristics should be considered in future studies.

For both species, we used submillimeter dMRI datasets to delineate bundles that would be inaccessible at lower resolution [90]. Our tractography pipeline was informed by an extensive evaluation of different dMRI acquisition and analysis strategies by comparing dMRI tractography to tracer injections in the same macaque brains [91, 92]. However, reconstructing some anatomical connections identified in the tracer data was still challenging in the dMRI data. E.g., very few streamlines were identified connecting the amygdala and the LPFC in both species. NHP tracer data show amygdala projections traveling through the uncinate fasciculus to reach their targets in the ventrolateral prefrontal cortex [93, 94], with similar fiber organization in humans [95]. In both species, dMRI data also showed false positive connections with the contralateral hemisphere. Some of these contralateral connections identified using dMRI are likely caused by the proximity of the fornix to actual amygdala pathways, such as the stria terminalis ( < 700 μm). Studies trying to separate these bundles also reported the partial volume effects in their tract reconstructions [96, 97]. Finally, some divergencies may relate to evolutive differences across species. Novel ex vivo imaging techniques under development show promise for providing ground truth of long-range axonal projections in the human brain (please refer to [98]). However, no current method has the precision of NHP tracer studies. Therefore, using NHP dMRI data as an intermediate step between NHP tract tracing and human dMRI is essential for identifying technic-related differences and excluding misleading evolutionary assumptions [87].

## Conclusion and future perspectives

We showed that the amygdala targeted by neurofeedback based on autobiographical memory recall has monosynaptic connections with the ipsilateral SN and DMN. It also connects with the contralateral DMN through areas involved in memory recall (hippocampus and PHG). This anatomical description is the first step towards improved circuit-based mechanistic hypotheses to be tested in the future. E.g., neurofeedback protocols using deep brain or intracranial electrodes are possible in macaques [99] and humans [100]. If paired with EEG or fMRI scans, these protocols may provide dynamic information about the amygdala modulation of large-scale networks. In this case, the synaptic configuration, directionality, and strength provided here are valuable priors for causal models [101]. Connectivity-based neurofeedback [102] probing specific connections may also incorporate this anatomic information to overcome physiological noise that increases the correlation between unconnected areas [103]. Identifying disynaptic pathways also provides additional - or alternative - targets within the anatomical circuit to optimize neurofeedback for non-responders to the amygdala modulation. Finally, we focused on the ROIs extracted from neurofeedback studies involving autobiographical recall as a strategy to upregulate the amygdala. The same translational pipeline can be used to delineate the underlying anatomy of amygdala downregulation and the associated involvement of other large-scale networks in different neurofeedback protocols [8].

## Supplementary information

Supplement

## Data Availability

The NHP datasets generated during and/or analyzed during the current study are available from the corresponding author upon reasonable request. The human dMRI data is publicly available in the Dryad repository at 10.5061/dryad.nzs7h44q2.
