# Supplementary material for: Translation of monosynaptic circuits underlying amygdala fMRI neurofeedback training
Source: Neuropsychopharmacology. 2024 Aug 5;49(12):1839–50. doi: 10.1038/s41386-024-01944-w (PMC11473645; doi:10.1038/s41386-024-01944-w)
Supplement: Supplementary file 1 — Supplement [file 41386_2024_1944_MOESM1_ESM.pdf]

SUPPLEMENTARY INFORMATION

Translation of monosynaptic circuits underlying amygdala fMRI neurofeedback training

Lucas Trambaiolli[1,2], Chiara Maffei[3], Evan Dann[3], Claudinei Biazoli Jr[4,5], Gleb Bezgin[6], Anastasia Yendiki[3], Suzanne Haber[1,2]

Methods

Anatomical injections

We identified the stereotaxic coordinates for the injection sites using pre-surgery structural MR images. Monkeys received injections of one or more of the following tracers: Lucifer Yellow (LY), Fluororuby (FR), Fluorescein (FS) (40 –50 nl, 10% in 0.1 M phosphate buffer [PB], pH 7.4; Invitrogen), or tritiated amino acids (100 nl, 1:1 solution of [3 H] leucine and [3 H]-proline in dH2O, 200 mCi/ml, NEN). Tracers were pressure-injected over 10 min using a 0.5 l Hamilton syringe. After each injection, the syringe remained *in situ* for 20-30 min.

Twelve to 14 days after the surgery, monkeys were deeply anesthetized and perfused with saline, followed by a 4% paraformaldehyde/1.5% sucrose solution. We post-fixed brains overnight and cryoprotected in increasing sucrose gradients [1]. We cut serial sections of 50 mm on a freezing microtome, and processed one in every eight free-floating sections to visualize LY, FR, FS, and AA tracers, as previously described [2,3]. We mounted sections onto gel-coated slides, dehydrated, defatted in xylene overnight, and coverslipped with Permount. In cases with more than one tracer injection into a single animal, we processed adjacent sections for each antibody reaction.

Retrograde charting used the StereoInvestigator software (MicroBrightField Bioscience, U.S.A), and anterograde charting used Neurolucida software (MicroBrightField).

NHP dMRI acquisition and preprocessing

NHP samples were packed in a bag, filled with fomblin, and occasionally manually massaged over two days to remove air bubbles trapped within the brain. We left the samples out at room temperature for a minimum of 6 hours prior to scanning. Samples were scanned in a small-bore 4.7T Bruker BioSpin MRI system, with a gradient internal diameter of 114 mm, maximum gradient strength 660 m/Tm, and a birdcage volume RF coil internal diameter of 72 mm. A two-shot 3D echo-planar imaging (EPI) sequence was

used for dMRI with TR = 500 ms, TE = 48 ms, δ = 15 ms, Δ = 19 ms, b_{max} = 40,000 s/mm^2, 514 gradient directions, and 0.5 mm isotropic resolution. The above scanning parameters result in a total scan time of 47 hours for each NHP sample.

The dMRI data underwent a preprocessing pipeline that included denoising [4] and correction for Gibbs ringing [5], signal drift [6], eddy-currents [7], and bias fields [8]. We fit fiber orientation distribution functions (fODF) to the pre-processed data using multi-shell multi-tissue constrained spherical deconvolution (MSMT-CSD [9]) in MRtrix3 [8]. The D99 macaque atlas [10] was transformed to each individual brain after registering the D99 magnetization transfer ratio (MTR) template volume to the individual b=0 volume using the robust affine registration in FreeSurfer (mri_robust_register [11]). The left amygdala was extracted from the D99 atlas, binarized, and dilated by 2 voxels in MRtrix3 to include the surrounding white matter. We performed probabilistic tractography in MRItrix3 seeding in every voxel within this mask (350 seeds per voxel). The following tractography parameters were used: step-size = 0.25 mm, maximum angle threshold = 30˚, fODF peak threshold = 0.06, and maximum length = 150 mm.

Human dMRI acquisition and preprocessing

We used high-resolution diffusion MRI (dMRI) data from a publicly available and pre-processed dataset [12]. Briefly, data were acquired on the MGH-USC 3T Connectome Scanner at 0.76 mm isotropic resolution using an SNR-efficient simultaneous multi-slab imaging technique (gSlider-SMS) [13,14] across 9 2-hour sessions (max gradient amplitude: 180 mT/m; slew rate: 125 T/m/s, gSlider factor: 5, MB factor: 2, R: 3, TR/TE: 3500/75 ms, matrix: 290x288, PE: AP). 2808 dMRI volumes were acquired (144 b=0, 420 b=1000, 840 b=2500 s/mm^2, and their paired reversed PE volumes).

We fit fODFs to the preprocessed dMRI data using multi-shell multi-tissue constrained spherical deconvolution (MSMT-CSD) in MRtrix3. Cortical parcellations and subcortical segmentations were obtained from the T1 data using FreeSurfer [15-17]. The left amygdala was extracted from the segmentation, binarized, and inflated by 1 voxel. We performed probabilistic tractography in MRtrix3, seeding every voxel within the amygdala (100 seeds per voxel). The following tractography parameters were used: step-size = 0.38 mm, maximum angle threshold = 45°, maximum length = 150 mm. We mapped the ROI coordinates from the MNI space to the individual space using the registration procedures described in the NHP analysis.

Cross-species brain parcellation

The “Regional Map” (RM) parcellation was initially manually drawn on the F99 macaque brain template [18]. Later, it was deformed to surface- and volume-based

human MNI representations [18,19] using a landmark-based deformation defined by a set of major 150 sulci and gyri, along with functional activation patterns, considered homologous between the two species [20].

Results

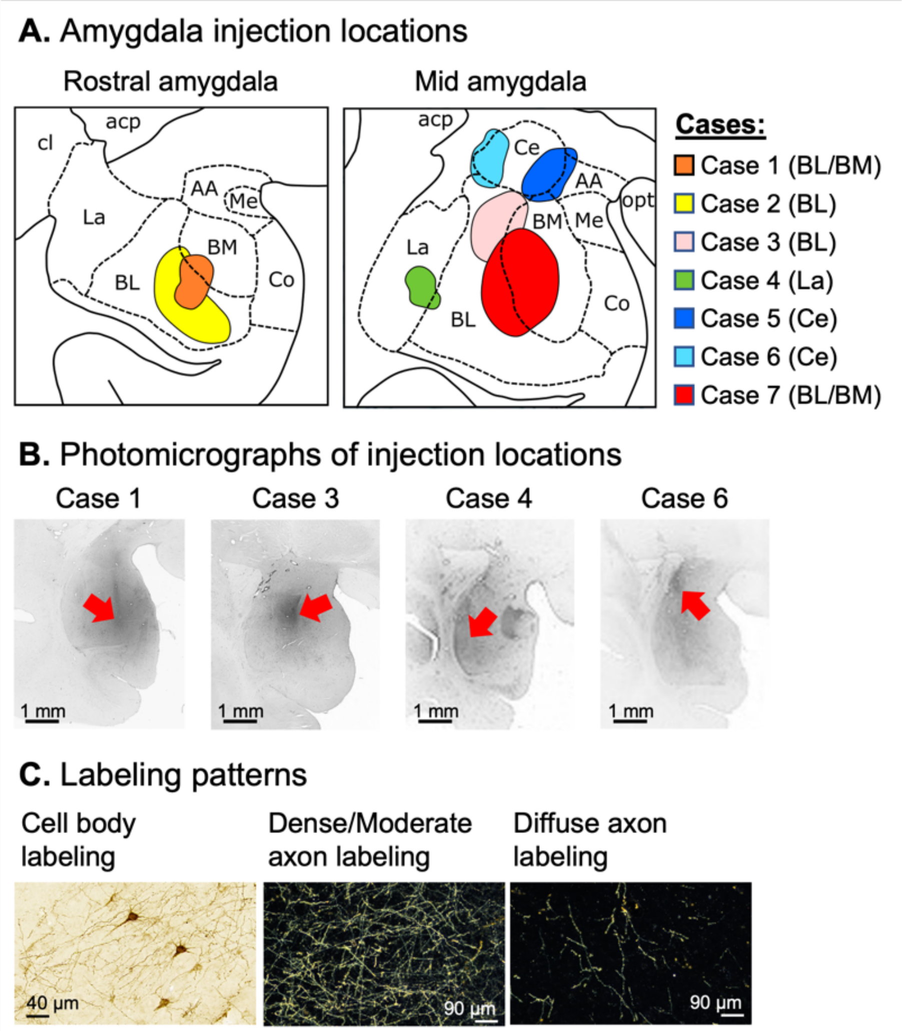

Supplementary Figure 1 – Amygdala Injection sites and labeling patterns. A. Schematic of injection sites at approximately the same rostro-caudal level in the macaque amygdala. Dotted lines = nuclei borders. Colored areas = individual cases. **B.** Coronal sections of the macaque amygdala showing different injection locations. Scale bar, 1 mm. **C.** Examples of dense/moderate and diffuse terminal fields. *Abbreviations*: AA = Anterior area; acp = anterior commissure - posterior limb; BL = Basolateral nucleus; BM = Basomedial nucleus; Ce = Central nucleus; cl = claustrum; Co = Cortical nucleus; La = Lateral nucleus; Me = Medial nucleus; opt = optic tract.

	Human brain					NHP brain				
	ROI coordinates (MNI space)			Morphological location [21]	Cytoarchitectonic location [22]	ROI coordinates (F99 space)			Morphological location	Cytoarchitectonic location [23]
	x	y	z			x	y	z		
**Ipsilateral (Left Hemisphere) Salience Network nodes**										
**Dorsal anterior cingulate cortex (dACC)**	-2	25	30	genu of the corpus callosum	24 and 32	-2	10	15	genu of the corpus callosum	24 and 6/32
**Lateral prefrontal cortex (LPFC)**	-50	14	0	border of frontal operculum and inferior frontal gyrus	44	-24	7	0	dorsal lip of the rostral part of lateral fissure	44, ProM, and 47O
**Anterior Insula (AI)**	-37	21	5	rostral and dorsal portions of circular sulcus	AI	-21	3	1	rostral and dorsal portions of circular sulcus	AI
**Ipsilateral (Left Hemisphere) Default Mode Network nodes**										
**Middle frontal gyrus**	-30	15	44	lateral portion of the superior frontal sulcus	6 and 8	-13	11	17	ventral bank of the superior arcuate sulcus	8AB, 8B, and 9/46D
**Temporal pole**	-43	3	-24	rostral and ventral portions of circular sulcus	Ipro and 22	-18	3	-6	rostral and ventral portions of circular sulcus	Ipro and TPPro
**Parahippocampal gyrus (PHG)**	-26	-4	-19	dorsal and medial to the rhinal sulcus	EOI, ELR	-8	-3	-14	dorsal and medial to the rhinal fissure	EOI, ELR, and ER
**Angular gyrus***	-44	-53	28	dorsal bank of the superior temporal sulcus, ascending posterior segment	39 and 22	-21	-23	15	caudal portion of the lateral fissure	PGOp, ReI, and TPt
**Medial precuneus***	-7	-59	40	ventral bank of the precuneal limiting sulcus	7 and 31	-1	-24	20	ventral bank of the posterior cingulate sulcus	PGm and 31
**Lateral precuneus**	-27	-54	46	lip of the ventral bank of the intraparietal sulcus	7 and 39	-14	-23	19	lip of the ventral bank of the intraparietal sulcus	POaE/LIPE and PG
**Contralateral (Right Hemisphere) Default Mode Network nodes**										
**Middle frontal gyrus**	28	45	33	ventral bank of the superior frontal sulcus	9 and 46	17	14	14	ventral bank of the principal sulcus	9/46V and 46V

**Temporal pole**	45	18	-29	dorsolateral portion of the anterior temporal lobe	TPCl	22	6	-9	dorsolateral portion of the anterior temporal lobe	TPPro and ST1
**Parahippocampal gyrus (PHG)**	30	-27	-23	medial wall of the collateral sulcus, parahippocampal ramus	TF and TH	17	-5	-18	lateral bank of the rhinal fissure	TLR/36R and TH
**Hippocampus**	30	-26	-2	mid portion of the hippocampus	CA1, CA2, CA3, DG	15	-12	-8	mid portion of the hippocampus	CA1, CA2, CA3, CA4
**Posterior cingulate cortex**	3	-47	31	posterior to the splenium of the corpus callosum	23 and 30	2	-23	11	posterior to the splenium of the corpus callosum	23 and 30
**Medial precuneus**	13	-31	50	dorsal bank of the posterior cingulate sulcus	4 and 31	6	-12	17	dorsal bank of the posterior cingulate sulcus	PECg and 31
**Thalamus**	17	-24	-2	mid portion of the thalamus	ventral, centromedial, mediodorsaln and pulvinar nuclei	9	-14	-1	mid portion of the thalamus	ventral, centromedial, mediodorsaln and pulvinar nuclei

Supplementary Table 1 – List of center or peak coordinates of nodes showing amygdala-rsFC changes when comparing pre- and post-amygdala neurofeedback training in human subjects and the equivalent coordinates in the homologous structures of the macaque brain. Nodes are grouped as part of the human Salience or Default Mode Networks. Coordinates in the human brain are reported in the MNI brain template (center), and coordinates in the macaque brain in the F99 brain template. * = coordinates estimated based on figures. *Abbreviations:* AI = Agranular insular cortex; ELR = Enthorinal cortex, lateral part, rostral division; EOI = Entorhinal cortex, olfactory part; ER = Entorhinal cortex, rostral part; IPro = Insular proisocortex; LIPE = Pateral intraparietal sulcus, external part; PECg= Parietal area PE, cingulate part; PG = Parietal area PG; PGM = Parietal area PG, medial part; PGOp = Parietal area PG, opercular part; POaE = Parieto-occipital associated area in the intraparietal sulcus, external part; ProM = Promotor area; Rel = Retroinsular cortex; ST1 = Superior temporal sulcus area 1; TF = Temporal area TF; TH = Temporal area TH; TLR = Temporal area TL, rostral part; TPCl = Temporoparietal cortex, lateral part; TPPro = Temporopolar proisocortex; TPt = Temporoparietal area.

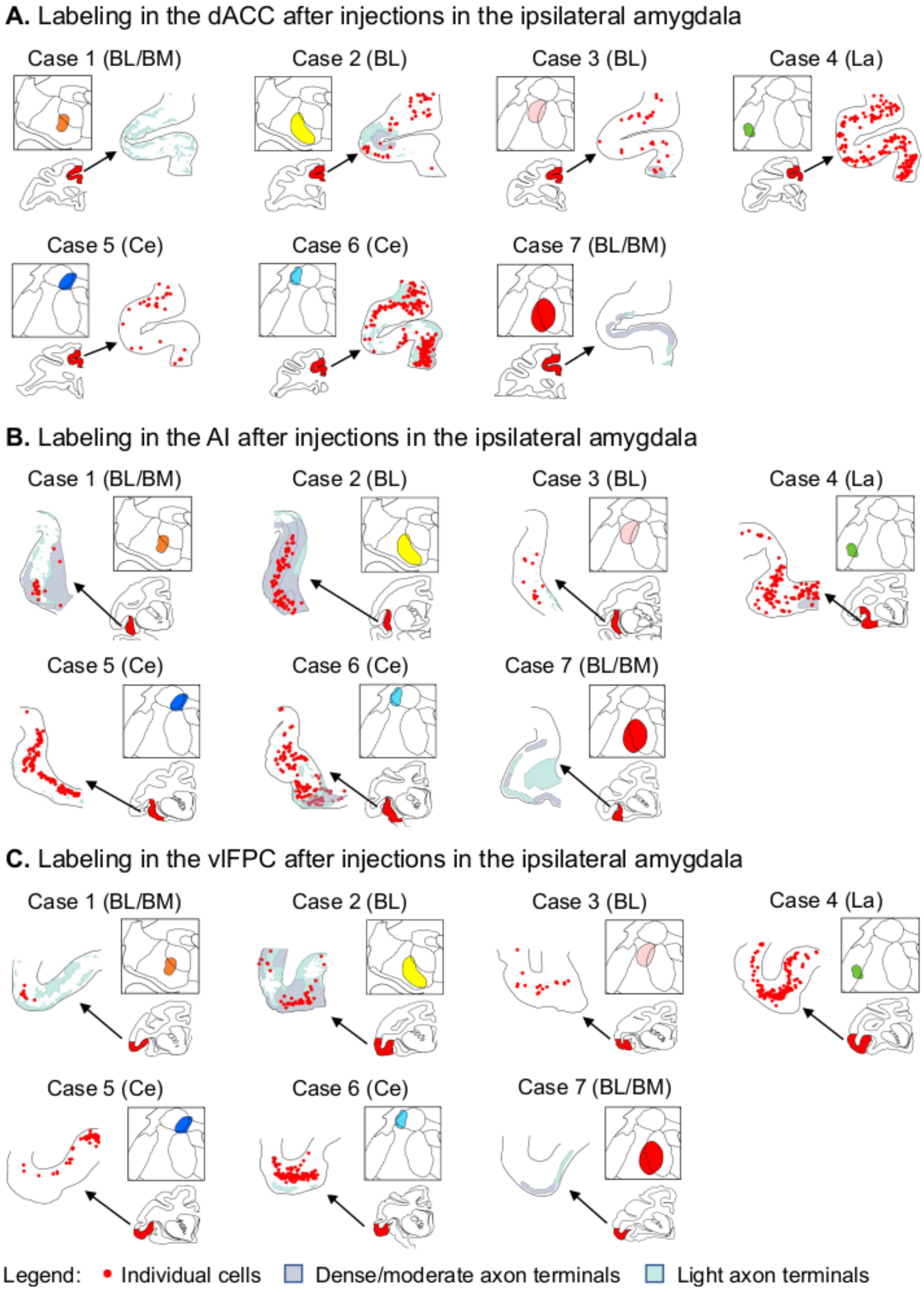
A. Labeling in the dACC after injections in the ipsilateral amygdala
Case 1 (BL/BM)
Case 2 (BL)
Case 3 (BL)
Case 4 (La)
Case 5 (Ce)
Case 6 (Ce)
Case 7 (BL/BM)
B. Labeling in the AI after injections in the ipsilateral amygdala
Case 1 (BL/BM)
Case 2 (BL)
Case 3 (BL)
Case 4 (La)
Case 5 (Ce)
Case 6 (Ce)
Case 7 (BL/BM)
C. Labeling in the vlFPC after injections in the ipsilateral amygdala
Case 1 (BL/BM)
Case 2 (BL)
Case 3 (BL)
Case 4 (La)
Case 5 (Ce)
Case 6 (Ce)
Case 7 (BL/BM)
Legend:
Individual cells
Dense/moderate axon terminals
Light axon terminals

Supplementary Figure 2 – Amygdala connections with the ipsilateral SN nodes. A) Each injection location is shown in the square box, and the schematic coronal sections highlight the dACC location with connectivity chartings in red. Individual cells are shown as red dots, dense/moderate terminals as light blue shaded areas, and diffuse terminals as light green shaded areas. The same organization followed for ROIs in the Temporal Pole (**B**), Parahippocampal Gyrus (**C**), Lateral Precuneus (**D**), Medial Precuneus (**E**), and Angular Gyrus (**F**). *Abbreviations:* BL = basolateral nucleus, BM = basomedial nucleus, C = caudal, Ce = central nucleus, La = lateral nucleus, R = rostral.

A. Labeling in the MFG after injections in the ipsilateral amygdala

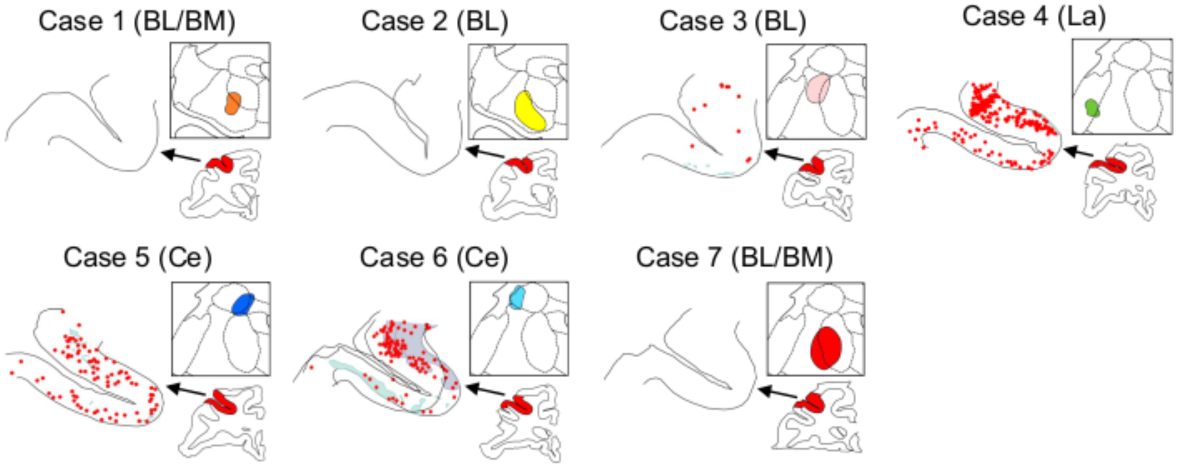

B. Labeling in the TP after injections in the ipsilateral amygdala

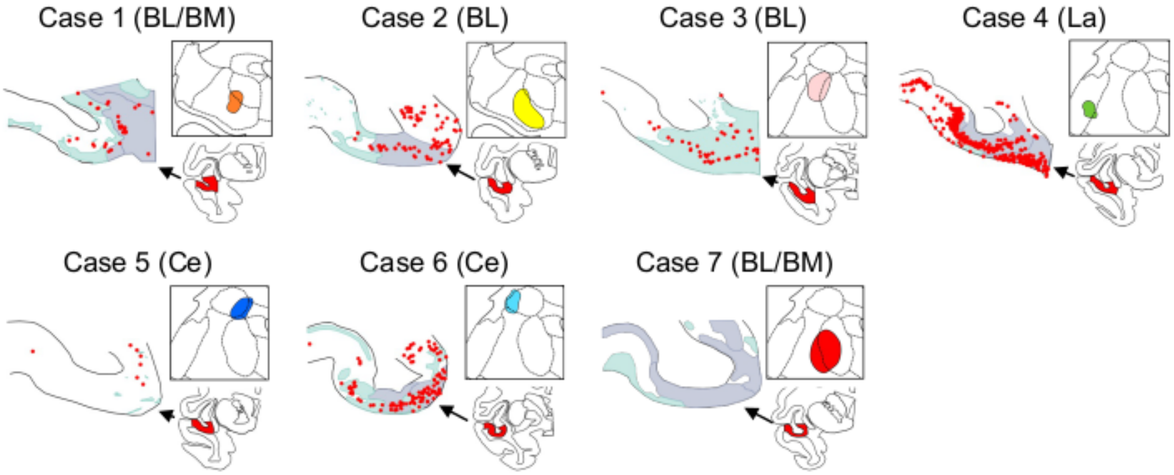

C. Labeling in the PHG after injections in the ipsilateral amygdala

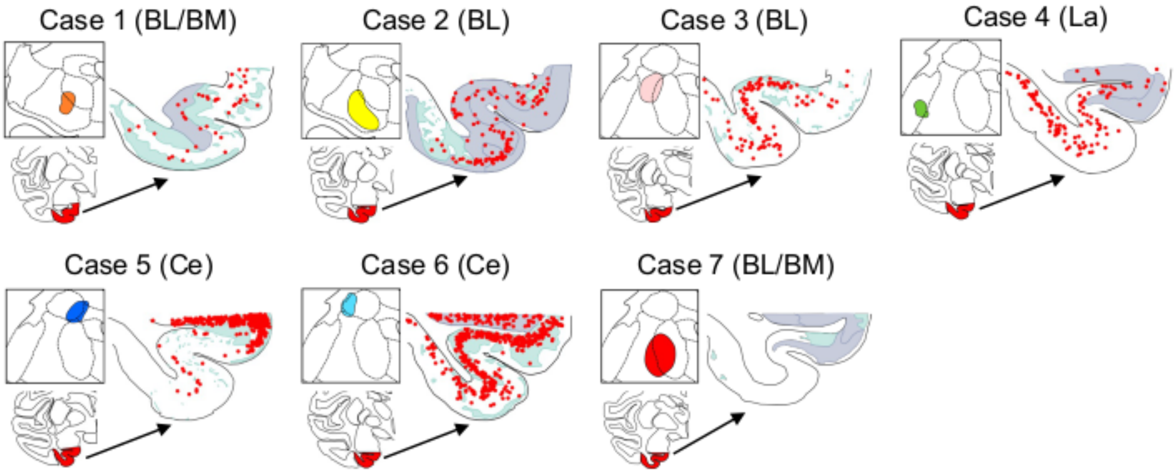

Legend: • Individual cells ▢ Dense/moderate axon terminals ▢ Light axon terminals

D. Labeling in the lateral Precuneus after injections in the ipsilateral amygdala

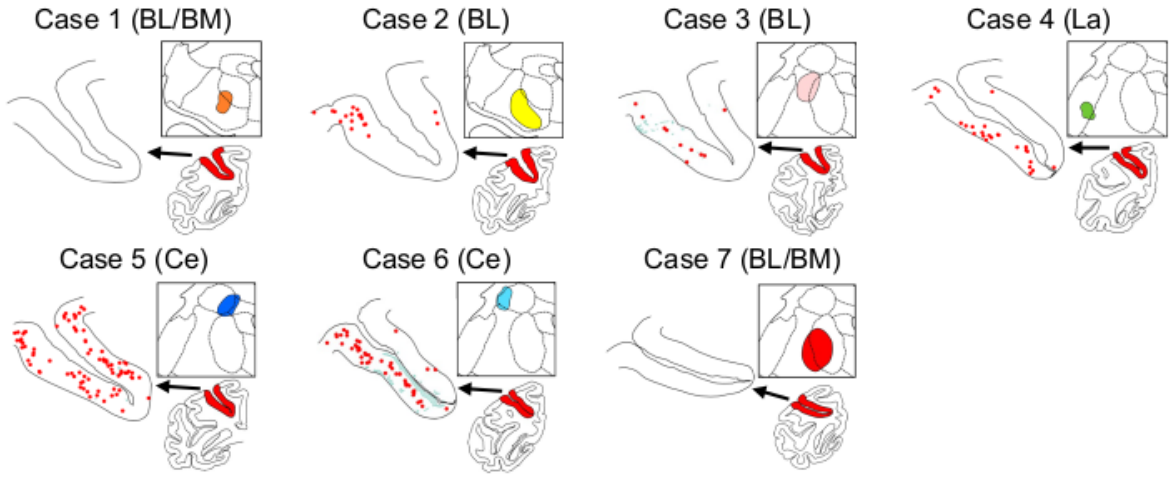

E. Labeling in the medial Precuneus after injections in the ipsilateral amygdala

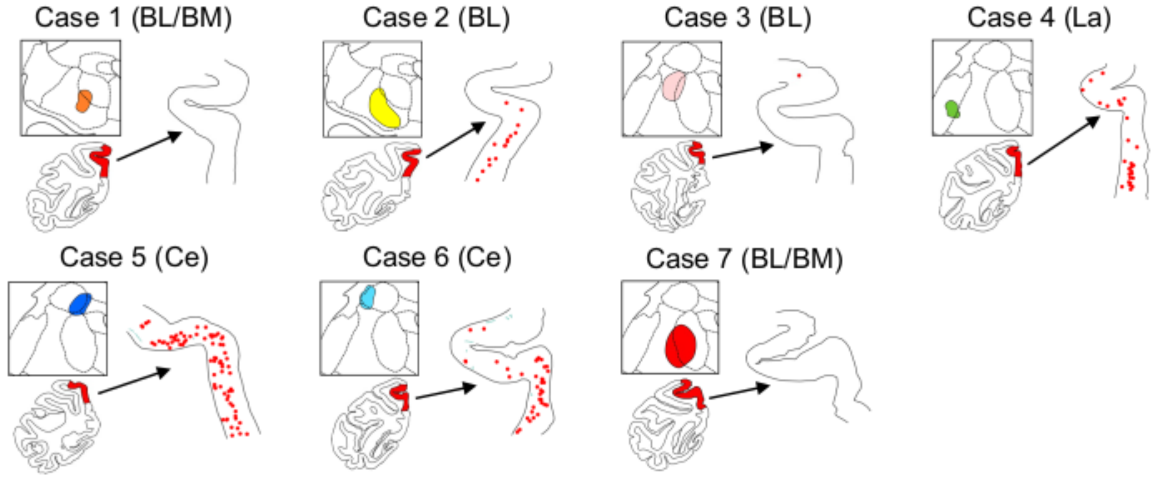

F. Labeling in the Angular Gyrus after injections in the ipsilateral amygdala

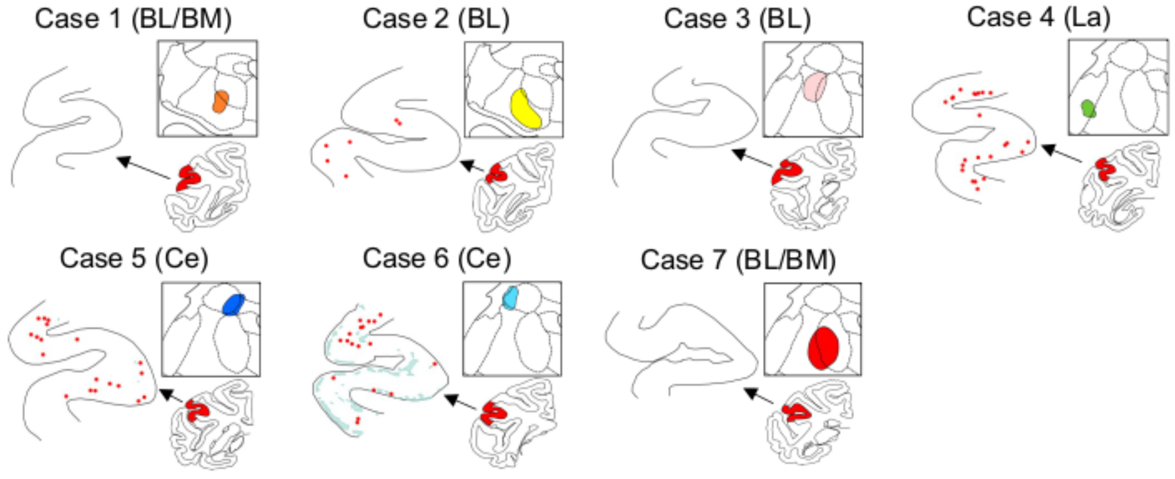

Legend: • Individual cells ▢ Dense/moderate axon terminals ▢ Light axon terminals

Supplementary Figure 3 – Amygdala connections with the ipsilateral DMN nodes. A) Each injection location is shown in the square box, and the schematic coronal sections highlight the MFG location with connectivity chartings in red. Individual cells are shown as red dots, dense/moderate terminals as light blue shaded areas, and diffuse terminals as light green shaded areas. The same organization followed for ROIs in the AI (**B**) and LPFC (**C**). *Abbreviations:* BL = basolateral nucleus, BM = basomedial nucleus, C = caudal, Ce = central nucleus, La = lateral nucleus, R = rostral.

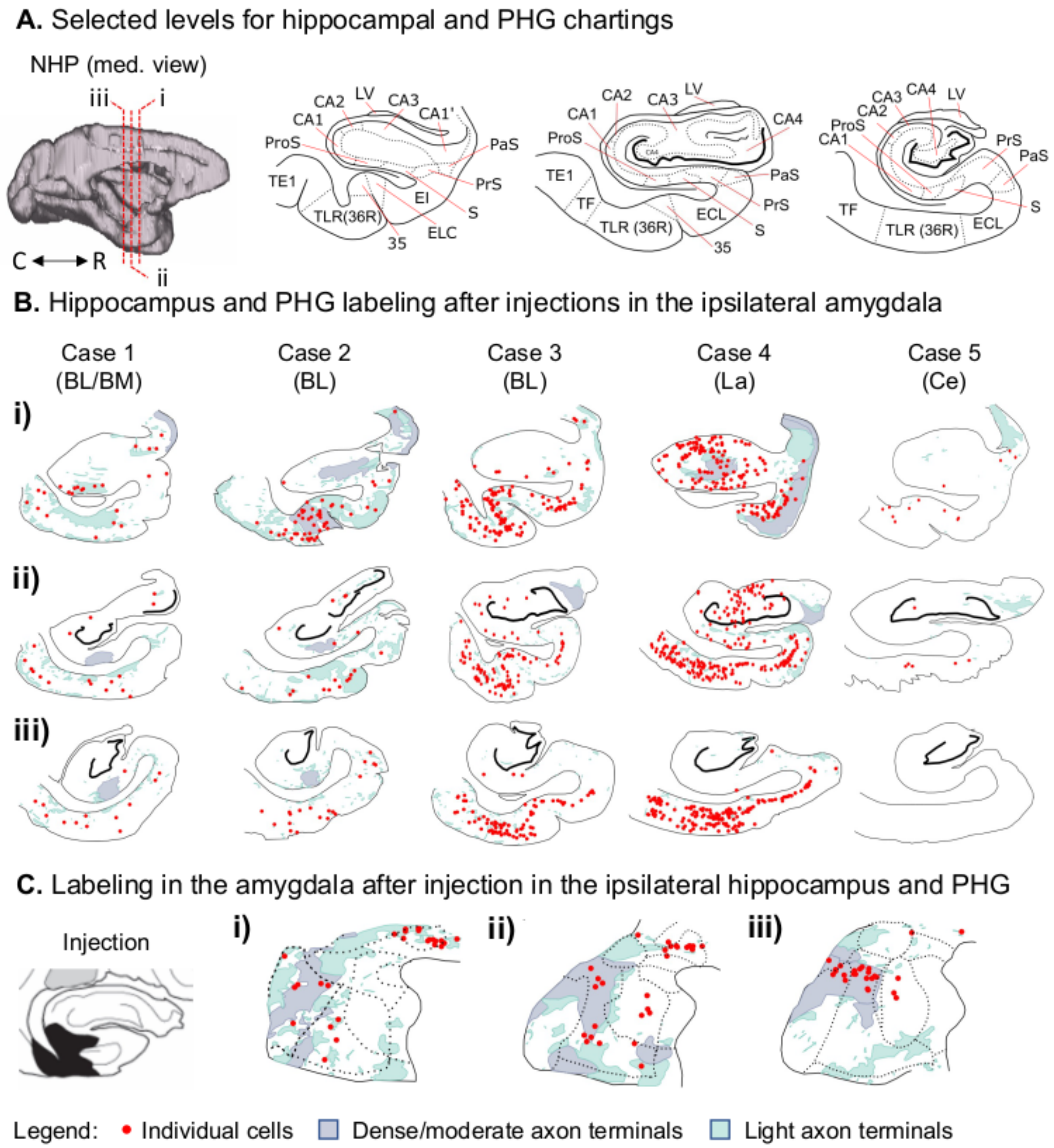

Supplementary Figure 4 – Amygdala connections with the hippocampus and parahippocampal gyrus (PHG). (**A**). 3D representation of the three rostro-caudal levels (a-b) used in the chart cells and terminals in the hippocampus and PHG, and the respective coronal slices with cytoarchitectonic divisions based on the Paxinos atlas [24]. Labeling of cells (red dots), and dense/moderate (light blue) and diffuse (light green) terminal fields in the hippocampus and PHG after bidirectional tracer injections in different amygdala nuclei (**B**). In (**C**), the labeling of cells and terminals in the amygdala (following the coronal levels a'-c' from Figure 5) after a bidirectional tracer injection in the hippocampus and PHG (left). *Abbreviations:* 35 = area 35 of cortex, BL = basolateral nucleus, BM = basomedial nucleus, C = caudal, CA1 = field CA1 of the hippocampus, CA1' = field CA1' of the hippocampus, CA2 = field CA2 of the hippocampus, CA3 = field CA3 of the hippocampus, CA4 = field CA4 of the hippocampus, Ce = central nucleus, ECL = caudal limit part of the entorhinal cortex, EI = intermediate part of the entorhinal cortex, ELC = lateral part of the entorhinal cortex, La = lateral nucleus, LV = lateral ventricle, PaS = parasubiculum, ProS = prosubiculum, PrS = presubiculum, R = rostral, S = subiculum, TE1 = temporal area TE1, TF = temporal area TF, TLR(36R) = rostral part of the temporal area TL (area 36R).

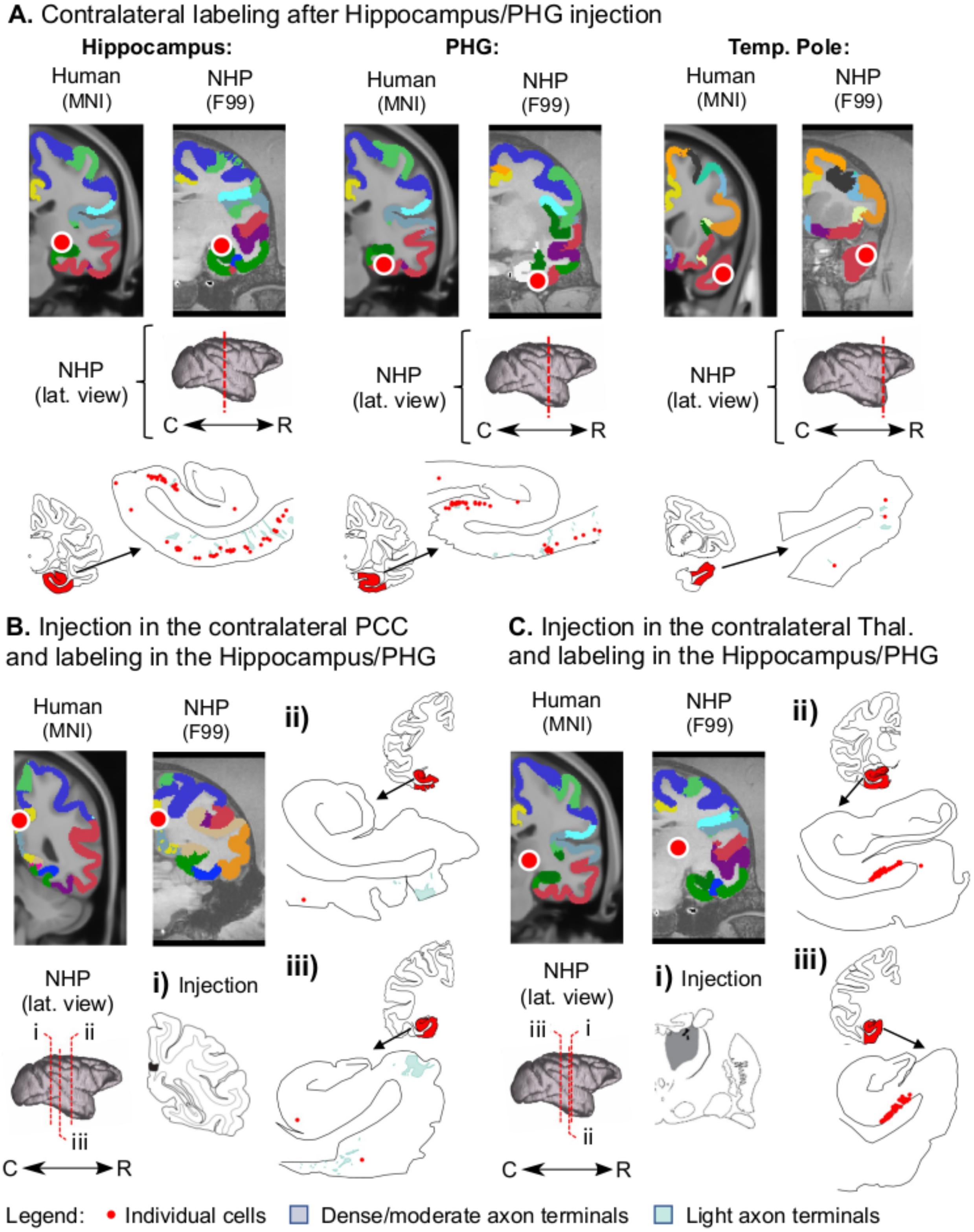
A. Contralateral labeling after Hippocampus/PHG injection
Hippocampus:
PHG:
Temp. Pole:
Human (MNI)
NHP (F99)
NHP (lat. view)
C
R
B. Injection in the contralateral PCC and labeling in the Hippocampus/PHG
C. Injection in the contralateral Thal. and labeling in the Hippocampus/PHG
i) Injection
ii)
iii)
Legend: Individual cells Dense/moderate axon terminals Light axon terminals

Supplementary Figure 5 – Hippocampus and parahippocampal gyrus (PHG) connections with contralateral DMN nodes. Red circles indicate the peak location of the rsFC changes after amygdala neurofeedback for the right Hippocampus (**A** left), PHG (**A** center), and Temporal Pole (**A** right). 3D models represent the rostro-caudal level from each node. For each region, schematic coronal sections highlight in red the location with connectivity chartings zoomed in showing labeling of cells (red dots) and dense/moderate (light blue) and diffuse (light green) terminal fields after bidirectional tracer injections in the contralateral Hippocampus and PHG (injection shown in Supplementary Figure 1C left). ROIs in the right PCC (**B**) and Thalamus (**C**) and respective injections also show labeled cells and terminals in the contralateral hippocampus and PHG. *Abbreviations:* BL = basolateral nucleus, BM = basomedial nucleus, C = caudal, Ce = central nucleus, La = lateral nucleus, R = rostral.

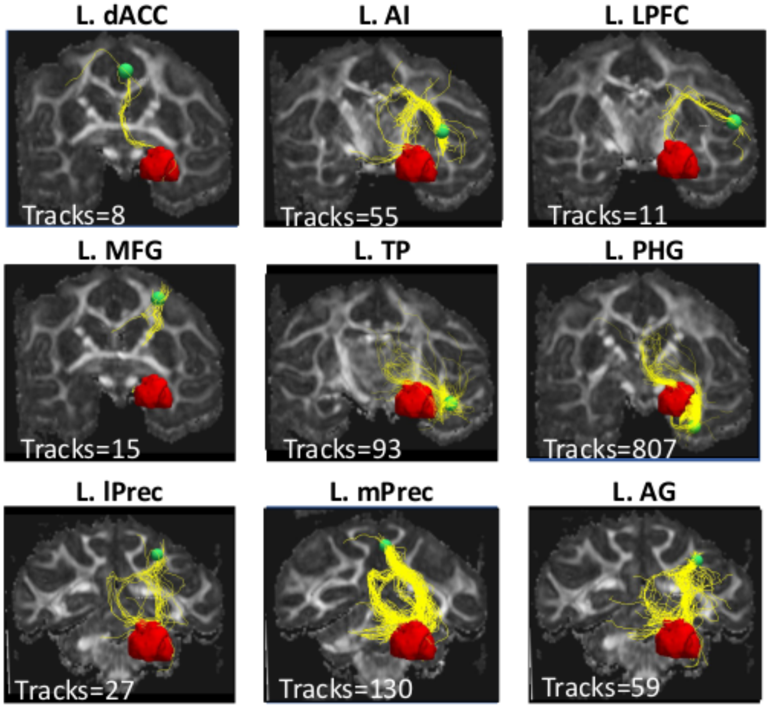

Supplementary Figure 6 – dMRI tractography results for animal 2. Examples of amygdala seeds (red) and the tracts (yellow) connecting them with all ipsilateral nodes (green) within the SN and DMN.

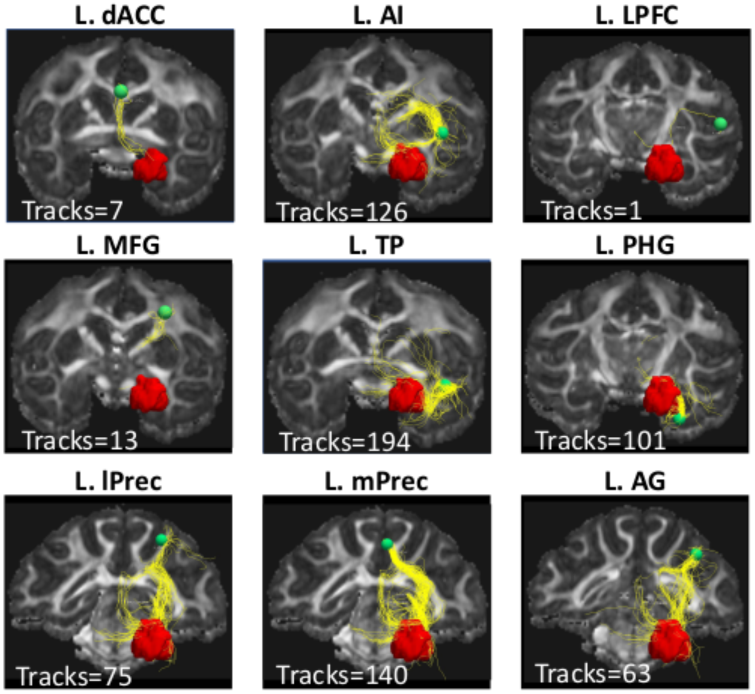

Supplementary Figure 7 – dMRI tractography results for animal 3. Examples of streamlines (yellow) connecting the amygdala (red) with all ipsilateral nodes (green) within the SN and DMN.

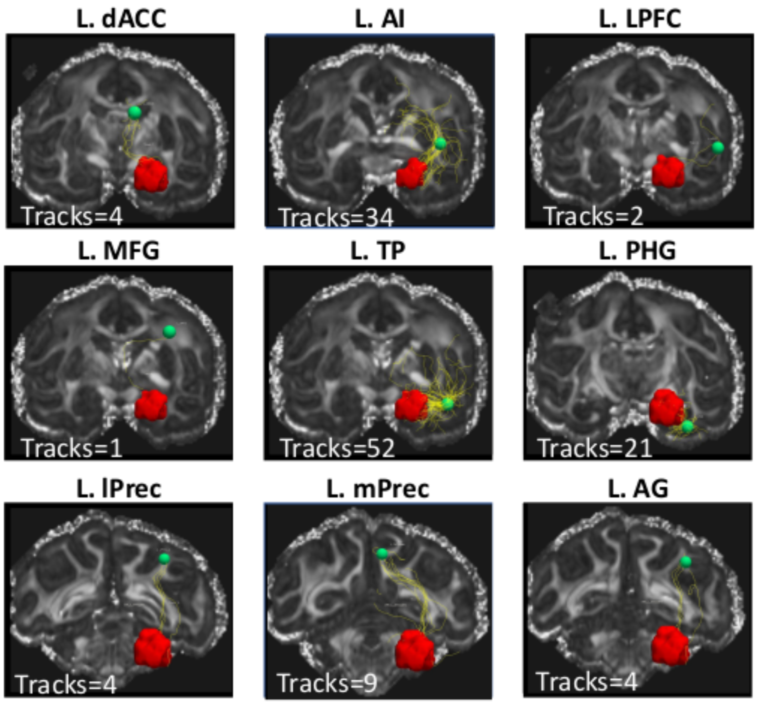

Supplementary Figure 8 – dMRI tractography results for animal 4. Examples of amygdala seeds (red) and the tracts (yellow) connecting them with all ipsilateral nodes (green) within the SN and DMN.

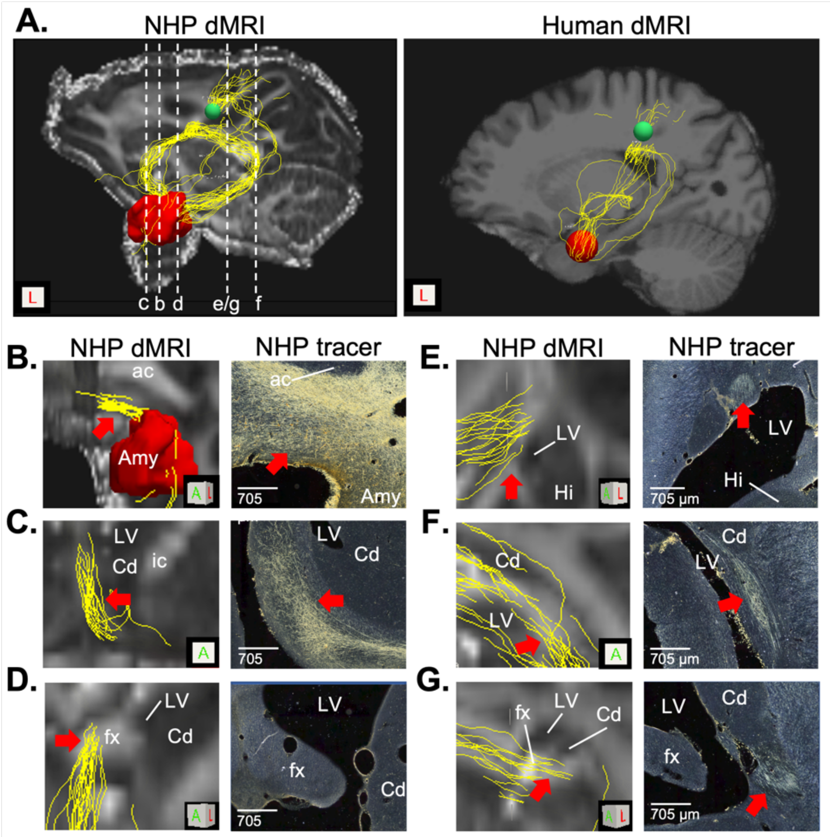

Supplementary Figure 9 – Tract tracing data allows for identifying false positive tractography results. (**A**) Results in the NHP and human brains show two major tracts (anterior and posterior) connecting the left amygdala and right medial precuneus. The most anterior tract follows the fibers of the amygdalofugal pathway from the amygdala (**B**) to the medial wall (**C**) until they are captured by the fornix (**D**). The posterior tract follows fibers from the stria terminalis (**E-G**) until they erroneously follow the fornix streamlines. *Abbreviations:* ac = anterior commissure, Amy = amygdala, cc = corpus callosum, Cd = caudate nucleus, fx = fornix, Hi = Hippocampus, ic = internal capsule, LV = lateral ventricle.

References

1 Haber SN, Kim KS, Mailly P, Calzavara R. Reward-related cortical inputs define a large striatal region in primates that interface with associative cortical connections, providing a substrate for incentive-based learning. J Neurosci. 2006;26(32):8368-76.

2 Haber SN, Kim K-S, Mailly P, Calzavara R. Reward-related cortical inputs define a large striatal region in primates that interface with associative cortical connections, providing a substrate for incentive-based learning. J Neurosci. 2006;26(32):8368-76.

3 Heilbronner SR, Haber SN. Frontal cortical and subcortical projections provide a basis for segmenting the cingulum bundle: implications for neuroimaging and psychiatric disorders. J Neurosci. 2014;34(30):10041-54.

4 Veraart J, Novikov DS, Christiaens D, Ades-Aron B, Sijbers J, Fieremans E. Denoising of diffusion MRI using random matrix theory. Neuroimage. 2016;142:394-406.

5 Kellner E, Dhital B, Kiselev VG, Reisert M. Gibbs-ringing artifact removal based on local subvoxel-shifts. Magnetic resonance in medicine. 2016;76(5):1574-81.

6 Vos SB, Tax CM, Luijten PR, Ourselin S, Leemans A, Froeling M. The importance of correcting for signal drift in diffusion MRI. Magnetic resonance in medicine. 2017;77(1):285-99.

7 Andersson JL, Graham MS, Zsoldos E, Sotiropoulos SN. Incorporating outlier detection and replacement into a non-parametric framework for movement and distortion correction of diffusion MR images. Neuroimage. 2016;141:556-72.

8 Tournier JD, Smith R, Raffelt D, Tabbara R, Dhollander T, Pietsch M, et al. MRtrix3: A fast, flexible and open software framework for medical image processing and visualisation. Neuroimage. 2019;202:116137.

9 Dhollander T, Mito R, Raffelt D, Connelly A. in *Proc Intl Soc Mag Reson Med* Vol. 555 (2019).

10 Saleem KS, Avram AV, Glen D, Yen CC-C, Frank QY, Komlosh M, et al. High-resolution mapping and digital atlas of subcortical regions in the macaque monkey based on matched MAP-MRI and histology. Neuroimage. 2021;245:118759.

11 Reuter M, Rosas HD, Fischl B. Highly accurate inverse consistent registration: a robust approach. Neuroimage. 2010;53(4):1181-96.

12 Wang F, Dong Z, Tian Q, Liao C, Fan Q, Hoge WS, et al. In vivo human whole-brain Connectom diffusion MRI dataset at 760 µm isotropic resolution. Scientific data. 2021;8(1):1-12.

13 Setsompop K, Fan Q, Stockmann J, Bilgic B, Huang S, Cauley SF, et al. High-resolution in vivo diffusion imaging of the human brain with generalized slice dithered enhanced resolution: Simultaneous multislice (g S lider-SMS). Magnetic resonance in medicine. 2018;79(1):141-51.

14 Wang F, Bilgic B, Dong Z, Manhard MK, Ohringer N, Zhao B, et al. Motion-robust sub-millimeter isotropic diffusion imaging through motion corrected generalized slice dithered enhanced resolution (MC-gSlider) acquisition. Magnetic resonance in medicine. 2018;80(5):1891-906.

15 Dale AM, Fischl B, Sereno MI. Cortical surface-based analysis. I. Segmentation and surface reconstruction. Neuroimage. 1999;9(2):179-94.

16 Fischl B, Sereno MI, Dale AM. Cortical surface-based analysis. II: Inflation, flattening, and a surface-based coordinate system. Neuroimage. 1999;9(2):195-207.

17 Fischl B, van der Kouwe A, Destrieux C, Halgren E, Segonne F, Salat DH, et al. Automatically parcellating the human cerebral cortex. Cereb Cortex. 2004;14(1):11-22.

18 Bezgin G, Vakorin VA, van Opstal AJ, McIntosh AR, Bakker R. Hundreds of brain maps in one atlas: registering coordinate-independent primate neuro-anatomical data to a standard brain. Neuroimage. 2012;62(1):67-76.
19 Bezgin G, Solodkin A, Bakker R, Ritter P, McIntosh AR. Mapping complementary features of cross-species structural connectivity to construct realistic “Virtual Brains”. Human brain mapping. 2017;38(4):2080-93.
20 Reid AT, Lewis J, Bezgin G, Khundrakpam B, Eickhoff SB, McIntosh AR, et al. A cross-modal, cross-species comparison of connectivity measures in the primate brain. Neuroimage. 2016;125:311-31.
21 Petrides M. Atlas of the morphology of the human cerebral cortex on the average MNI brain. Academic Press; 2018.
22 Mai J, Paxinos G, Voss T. Atlas of the Human Brain. Elsevier; 2008.
23 Paxinos G, Huang XF, Toga AW. The rhesus monkey brain in stereotaxic coordinates. Academic Press: San Diego, CA; 2000.
24 Paxinos G, Huang X-F, Toga AW. The rhesus monkey brain in stereotaxic coordinates. 2000.
